# Fluctuations in cell density alter protein markers of multiple cellular compartments, confounding experimental outcomes

**DOI:** 10.1371/journal.pone.0211727

**Published:** 2019-02-04

**Authors:** Katarina Trajkovic, Clarissa Valdez, Daniel Ysselstein, Dimitri Krainc

**Affiliations:** Department of Neurology, Northwestern University Feinberg School of Medicine, Chicago, Illinois, United States of America; Univerzitet u Beogradu, SERBIA

## Abstract

The life cycle of cultured proliferating cells is characterized by fluctuations in cell population density induced by periodic subculturing. This leads to corresponding changes in micro- and macroenvironment of the cells, accompanied by altered cellular metabolism, growth rate and locomotion. Studying cell density-dependent morphological, physiological and biochemical fluctuations is relevant for understanding basic cellular mechanisms and for uncovering the intrinsic variation of commonly used tissue culture experimental models. Using multiple cell lines, we found that expression levels of the autophagic markers p62 and LC3II, and lysosomal enzyme cathepsin D were altered in highly confluent cells as a consequence of nutrient depletion and cell crowding, which led to inactivation of the mTOR signaling pathway. Furthermore, both Lamp1 and active focal adhesion kinase (FAK) were reduced in high-density cells, while chemical inhibition or deletion of FAK led to alterations in lysosomal and autophagic proteins, as well as in the mTOR signaling. This was accompanied by alterations in the Hippo signaling pathway, while cell cycle checkpoint regulator p-cdc2 remained unaffected in at least one studied cell line. On the other hand, allometric scaling of cellular compartments in growing cell populations resulted in biochemically detectable changes in the plasma membrane proteins Na^+^K^+^-ATPase and cadherin, and nuclear proteins HDAC1 and Lamin B1. Finally, we demonstrate how treatment-induced changes in cell density and corresponding modulation of susceptible proteins may lead to ambiguous experimental outcomes, or erroneous interpretation of cell culture data. Together, our data emphasize the need to recognize cell density as an important experimental variable in order to improve scientific rigor of cell culture-based studies.

## Introduction

*In vitro* cell maintenance entails permanent cycling of cell populations between sparse and confluent states which occurs between passages. This cycling leads to continuous readjustment of cells to fluctuating conditions at individual and cell population levels. For instance, growing cell populations gradually exhaust available nutrients from the media, which in turn leads to altered cellular proteostasis and metabolism [1–4]. Progressive loss of available space between cells leads to a decrease in cell surface area adhering to extracellular matrix, which affects cell size [5] and the number of focal adhesions, as well as the associated focal adhesion kinase (FAK) signaling [6]. A concomitant increase in intercellular contacts and E-cadherin homophilic binding at the cell surface stimulate Hippo signaling pathway leading to contact inhibition of proliferation which prevents unlimited population growth [7–9]. On a single-cell level, local crowding-dependent changes in FAK signaling create cell-to-cell variability in lipid composition [10, 11]. Moreover, seemingly unrelated processes such as endocytosis and viral infection depend on the local crowding and population context of each individual cell [12].

Cell density-dependent events have received much attention in the context of physiological and pathophysiological processes. Contact inhibition has been extensively studied in development and the cancer field [13–15], and its implications for aging emerged from a study which showed that high cell density suppresses cell senescence program through mTOR pathway inactivation [16]. However, despite the mounting evidence showing the influence of cell density on cellular biochemistry and physiology, little systematic research has been done on fluctuating cell density as a source of variability in specific protein expression levels in tissue culture experimental systems.

In this study, we found that autophagic proteins and lysosomal enzyme cathepsin D respond to variation in cell confluency due to the accompanying changes in nutrient availability and cell crowding, both of which act at least partially through mTOR-signaling pathway. In addition, in HEK293FT cells, local crowding regulates cell-to-cell variability in abundance of lysosomal marker Lamp1 in a FAK-dependent manner. This was accompanied by changes in Hippo pathway in all analyzed cell lines, but not in cell cycle dynamics in at least one cell line. Furthermore, allometric scaling of cellular compartments associated with increasing cell density leads to biochemically detectable shifts in multiple plasma membrane and nuclear markers, such as Na^+^K^+^-ATPase, cadherin, HDAC1 and Lamin B1. Finally, we demonstrate how adaptation of selected proteins to cell population density may affect results of Western blot analysis, and suggest a rational experimental design to minimize the impact of cell confluency as an undesired variable in cell culture experiments.

## Results

### Cell density affects the expression levels of autophagic and lysosomal proteins

**Fig 1:**
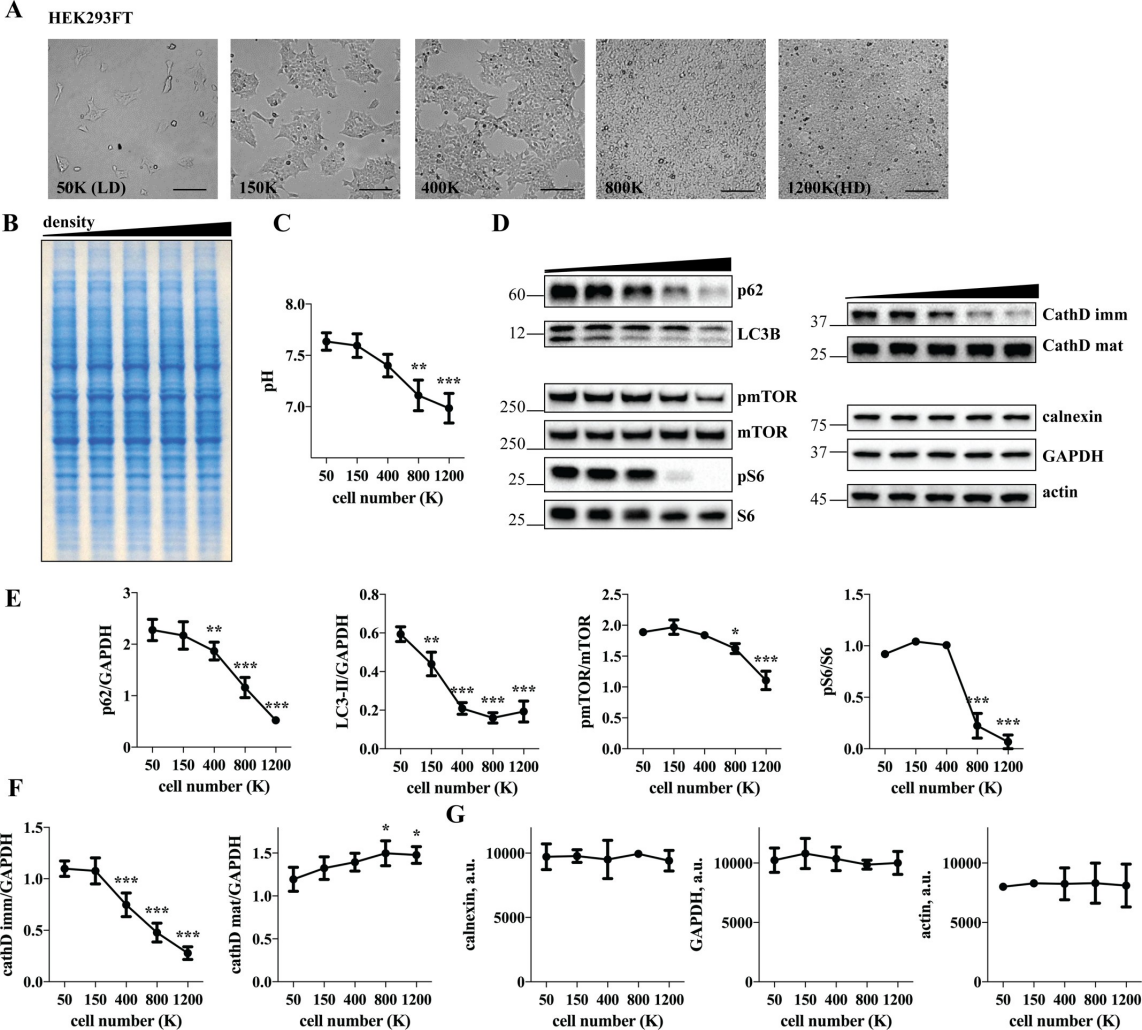
Autophagic proteins, mTOR signaling and cathepsin D are sensitive to cell confluence in HEK 293FT cells. (A) HEK 293FT cells plated at a range of densities were incubated for two days and imaged by light microscopy. The number of plated cells is indicated in the lower left corner of each image. LD, low density; HD, high density; scale bar 100 μm. (B) cells were lysed and equal amounts of proteins were separated by SDS PAGE, followed by visualization of the proteins by SimplyBlue; (C) pH of the media was determined before the cell lysis; (D) Cell lysates were analyzed by Western blotting using indicated antibodies; (E-G) Western blot images were quantified and the values normalized to GAPDH, unless indicated otherwise. N = 3, except for p62, cathD mat (N = 4) and cathD imm (N = 5); Line graph data are mean ± SD. *p<0.05, **p<0.01, ***p<0.001, relative to 50K cells.

We observed a remarkable variability in the expression levels of several proteins as a function of cell confluency which prompted us to address this issue systematically, by growing multiple cell lines–HEK 293FT, A431, HeLa and MEF at a range of densities from low to high (Fig 1A, S1–S3 Figs). To assess the magnitude of density-dependent changes, total cellular proteins separated on polyacrylamide gels were visualized by SimplyBlue unspecific protein dye and no obvious perturbations in major protein bands were detected (Fig 1B; S1–S3 Figs). However, a significant drop in pH in highly confluent cells (Fig 1C; S1–S3 Figs) was indicative of lactic acid secretion, possibly due to metabolic changes within the cells. Therefore, we performed Western blot analysis of candidate proteins involved in metabolic functions such as nutrient-sensing and autophagy. A striking response to an increase in cell density was observed for a nutrient-sensor [17] and autophagic substrate [18] p62, and autophagosomal marker LC3B (Fig 1D and 1E; S1 and S2 Figs). Since mTOR is one of the major regulators of cellular metabolism [19], the activity of mTOR signaling pathway was assessed by monitoring Ser2448 phosphorylation of mTOR [20] and the levels of mTOR activity marker pS6 across cell density gradients. A steep decline in the levels of pS6 in high density conditions was observed, as previously reported [16], as well as a reduction in pSer2448 mTOR (Fig 1D and 1E; S1–S3 Figs). Since nutrient depletion also regulates transcriptional activation of lysosomal genes through mTOR/TFEB regulatory axis [21, 22], we next analyzed the expression levels of a lysosomal protease cathepsin D and found a direct correlation between cell density and the levels of mature cathepsin D. Interestingly, the immature forms of the enzyme exhibited variable, cell type-specific patterns (Fig 1D and 1F; S1–S3 Figs), likely because their levels depend on the combined effect of *de novo* protein synthesis and cleavage, both of which may be affected by variations in cell density. However, highly abundant cytosolic (GAPDH), cytoskeletal (beta actin) and endoplasmic reticulum membrane (calnexin) markers were stable across all tested cell densities (Fig 1D and 1G; S1–S3 Figs), showing that the adaptation to cell confluency was limited to a subset of cellular proteins.

### Cell density-dependent change in autophagic proteins is regulated by media conditioning and cell crowding via inactivation of mTOR signaling pathway

**Fig 2:**
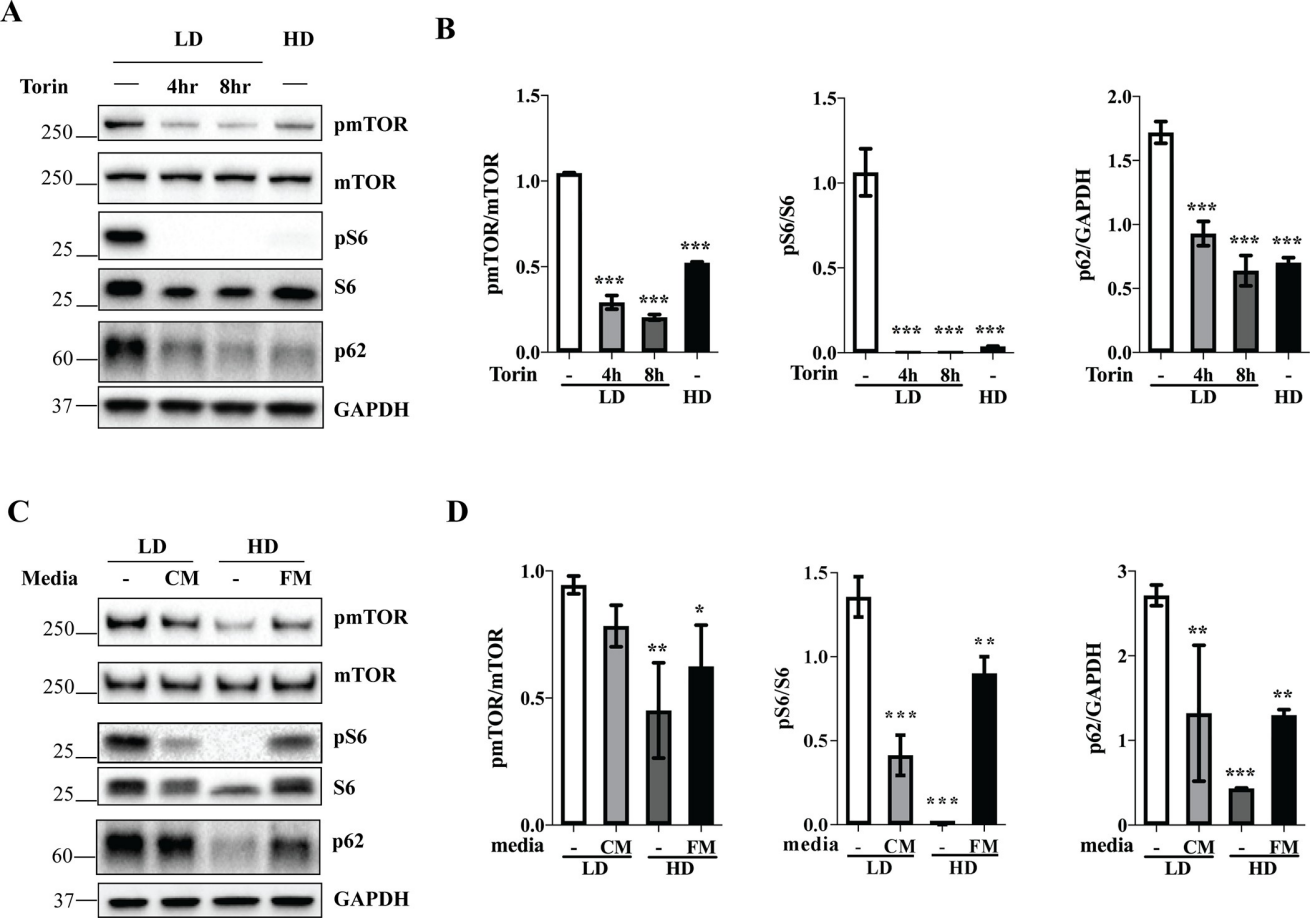
Media conditioning and cell crowding regulate autophagy through inactivation of mTOR signaling pathway. (A) HEK 293FT cells were plated at low density (LD, 50K) or high density (HD, 1200K) and treated with vehicle (-) or 330 nM Torin for the indicated period of time before cell lysis. (C) Cells were plated at low or high density and incubated during the entire experiment in intact media (-), conditioned media (CM), or in the media with continuously maintained freshness (FM), as indicated. (A,C) Cell lysates were analyzed by Western blotting using indicated antibodies. (B,D) Western blot images were quantified and the values normalized to GAPDH unless indicated otherwise. N = 3; Bar graph data are mean ± SD. *p<0.05, **p<0.01, ***p<0.001, relative to LD-.

To test whether mTOR signaling was responsible for the adaptation of autophagic/lysosomal proteins to cell density, we interfered with mTOR function in low-density HEK 293FT cells using Torin 1 and found that mTOR inhibition phenocopied high-density condition in terms of p62 and pS6 downregulation (Fig 2A and 2B). The role of media exhaustion in triggering mTOR inactivation was assessed by incubating low-density cells with the media collected from high-density cells. However, mTOR inactivation, as well as the alteration in autophagic markers were only partially recapitulated under these conditions (Fig 2C and 2D), which was indicative of the involvement of additional factors. Since cellular crowding also triggers an adaptive response through various mechanisms including contact inhibition [23], an inverse experiment was performed, where high-density cells were incubated in maximum volumes of the media, with periodical replacement of the media to maintain freshness. This approach revealed that cellular crowding in nutrient-rich conditions was sufficient to trigger downregulation of pS6 and p62, albeit not to the same extent as in high-density cells with intact media (Fig 2C and 2D). Together, these experiments suggest that media exhaustion and cellular crowding regulate mTOR pathway synergistically upon increase in cell density.

### Cell crowding regulates lysosomes, autophagy and mTOR signaling in a FAK-dependent manner

**Fig 3:**
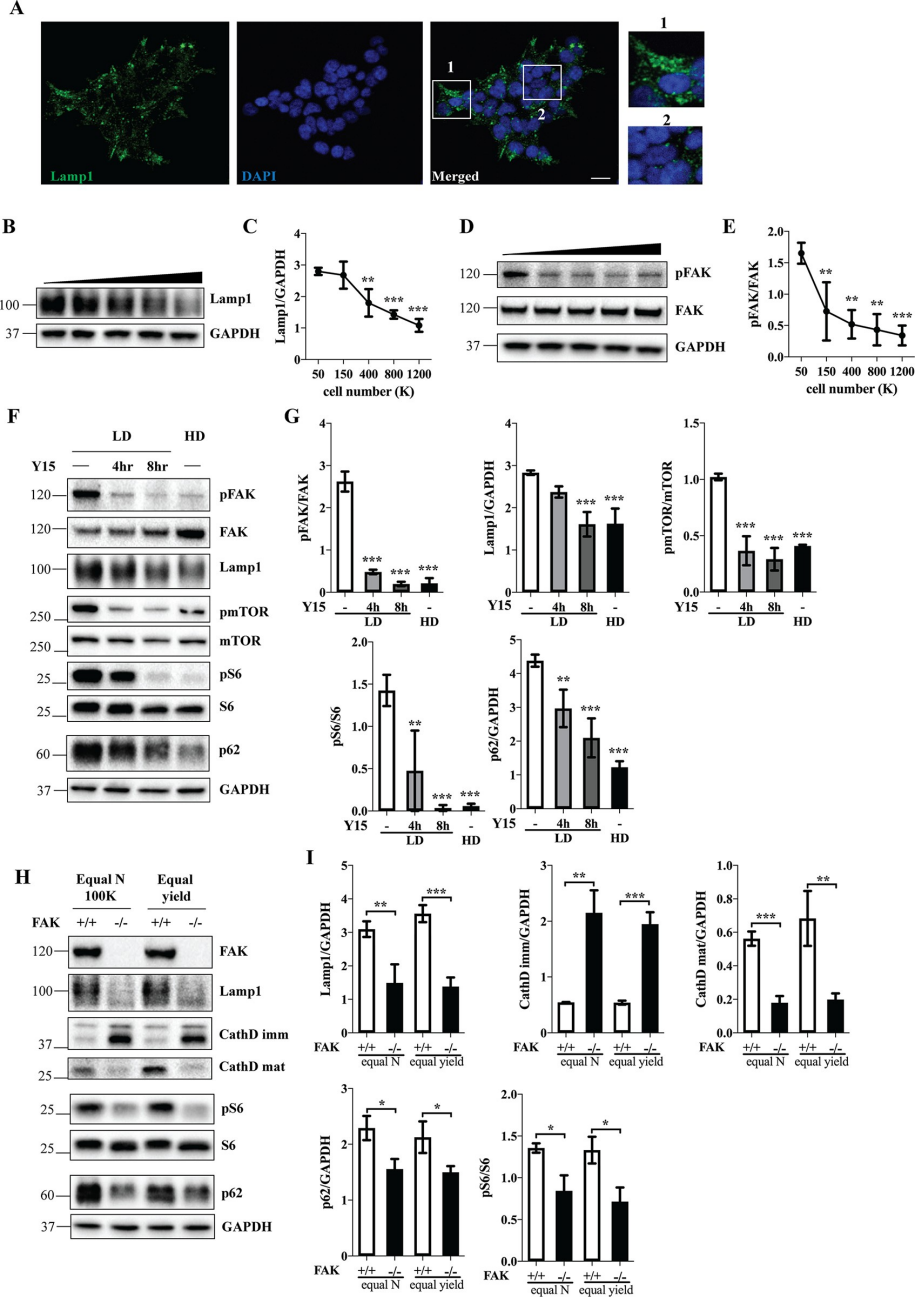
Cell crowding regulates lysosomes, autophagy and mTOR signaling in a FAK-dependent manner. (A) HEK 293FT cells were plated at 100K on coverslips placed in a 6 well plate, incubated for 2 days, fixed and stained against Lamp1; DAPI was used to visualize nuclei. 1, edge cell; 2, non-edge cell. Scale bar, 20 μm. (B,D) HEK 293FT cells were plated at a range of densities and incubated for two days. Cell lysates were analyzed by Western blotting using indicated antibodies. GAPDH was used as a loading control. (F) Cells were plated at low (LD) or high (HD) density and treated with the vehicle (-) or 25 μM FAK inhibitor Y15 for the indicated period of time before cell lysis. Cell lysates were analyzed by Western blotting using indicated antibodies. (H) FAK+/+ and FAK-/- MEF cells were plated at a range of densities, incubated for two days and lysed in RIPA buffer. First set: cells plated at the same number (Equal N, 100K); second set: cells with matching protein yield at the end of experiment (Equal yield). Cell lysates were analyzed by Western blotting and probed with the indicated antibodies. (C,E,G,I) Western blot images were quantified and the values normalized to GAPDH unless indicated otherwise. N = 3; Line and bar graph data are mean ± SD. *p<0.05, **p<0.01, ***p<0.001, relative to 50K (C,E) or LD–(G).

Since subconfluent HEK 293FT cells form relatively segregated colonies, we took advantage of this uneven cell distribution to visually monitor the effects of cell crowding on autophagy/lysosomal system. Interestingly, a remarkable contrast was observed between the cells at the edge of a colony and centrally positioned cells, the latter displaying a reduction in late-endosomal/lysosomal marker Lamp1 (Fig 3A and S4 Fig). Moreover, Western blot analysis showed that Lamp1 levels were inversely correlated with cell confluency (Fig 3B and 3C), likely because the ratio of edge to non-edge cells in the total cell population progressively decreases as the colonies expand [12]. However, in A431 cells, Lamp1 levels were unaffected by increasing cell density and immunofluorescence revealed relatively uniform Lamp1 intensity regardless of the cell position within a colony (S5 Fig), suggesting that cell-to-cell variability in Lamp1 may be a cell type-specific adaptation to population context. Attenuated mTOR signaling in high-density cells is unlikely to explain the loss of Lamp1 compartment, since the concomitant activation of TFEB would lead to an increase in transcription of lysosomal genes, including Lamp1 [24]. On the other hand, inactivation of FAK through Tyr397 dephosphorylation is the major trigger for local crowding-dependent alterations in cellular lipid composition [10]. We hence examined the potential role of FAK signaling in cell density-dependent Lamp1 downregulation. Western blot analysis revealed a decrease in pFAK/FAK ratio with increasing cell density (Fig 3D and 3E). Moreover, when low-density cells were treated with FAK inhibitor Y15, the levels of Lamp1 were decreased similarly to those in high-density cells (Fig 3F and 3G). Interestingly, chemical inhibition of FAK also led to a decrease in pmTOR, as well as to a reduction in pS6 and p62 (Fig 3F and 3G), suggesting that diminished FAK activity may act in synergy with nutrient deficiency to silence mTOR-signaling pathway and activate autophagy in high-density cells. The involvement of FAK in the evolution of high-density cellular phenotypes was corroborated by comparing MEF cells obtained from FAK knockout mice to those from their wild-type littermates. The two lines manifested different cell size, shape and growth rate. To account for these differences, FAK +/+ and FAK -/- cells were plated at a range of densities and matched for comparison either based on equal number of plated cells, or on equal protein yield at the end of the experiment (Fig 3H). In both cases, FAK -/- cells displayed a significant decrease in the levels of Lamp1, p62 and pS6 (Fig 3H and 3I), consistent with the phenotypes observed in HEK 293FT cells upon FAK inhibition (Fig 3F and 3G) and in high-density MEFs (S3 Fig). In addition, while the immature cathepsin D forms were increased, the mature form was decreased, suggesting the impairment of cathepsin D processing upon FAK deletion. (Fig 3H and 3I).

### Altered Hippo signaling, but not cell cycle dynamics, accompanies cell density-dependent changes

**Fig 4:**
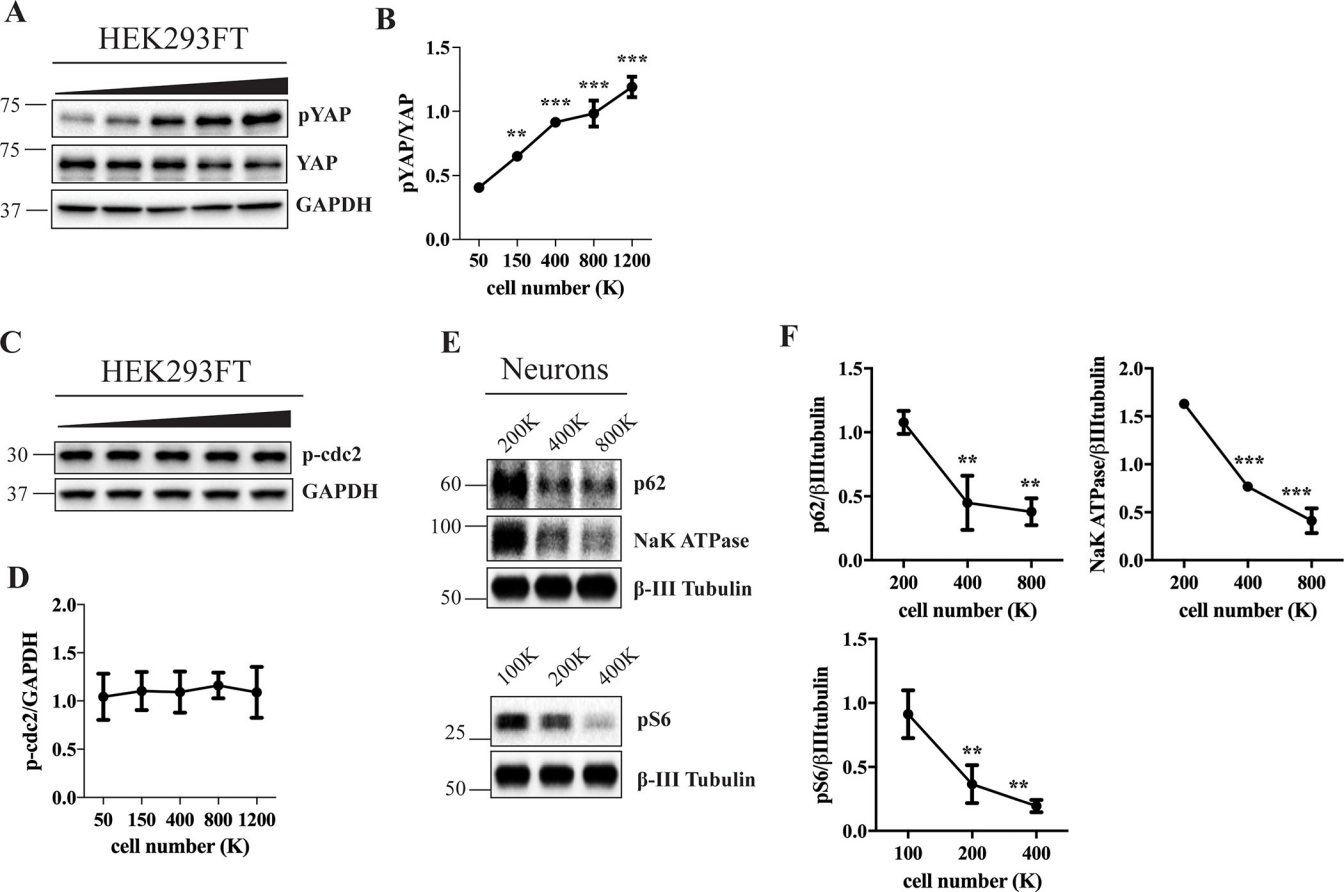
Hippo signaling depends on cell density without accompanying changes in cell cycle dynamics in HEK 293FT cells. (A, C) HEK 293FT cells were plated at a range of densities and incubated for two days. Cell lysates were analyzed by Western blotting using indicated antibodies. GAPDH was used as a loading control. (E) Neuronal precursors were plated at a range of densities and differentiated for 35–43 days. Cell lysates were analyzed by Western blotting using indicated antibodies. β-III Tubulin was used as a loading control. (B, D, F) Western blot images were quantified and the values normalized to total YAP (B), GAPDH (D) or β-III Tubulin (F). N = 3; Line graph data are mean ± SD. **p<0.01, ***p<0.001, relative to 50K (B), 200K (F, top) or 100K (F, bottom).

So far, our data show that cell density-dependent nutrient availability and cell crowding affect mTOR and FAK signaling pathways, which in turn modulate the expression of autophagic and lysosomal proteins. On the other hand, fluctuating cell density in tissue culture conditions is also coupled with alterations in Hippo signaling and cell cycle progression [9]. To assess Hippo signaling under different cell densities in our experimental models, we quantified by Western blot analysis the expression levels of pYAP, a central mediator of the Hippo pathway and found a direct correlation between pYAP/YAP ratio and cell density in all cell types (Figs 4A and 3B and S6 Fig), as expected [9]. To correlate cell cycle dynamics with the observed phenotypes, we analyzed the levels of Y15-phosphorylated cell cycle checkpoint regulator cdc2 in cell density gradients of HEK 293FT, MEF and HeLa cells. However, while p-cdc2 was inversely correlated with cell density in MEF and HeLa cells (S7 Fig), it remained stable in HEK 293FT cells (Fig 4C and 4D), suggesting that the cell cycle is not a critical factor in the regulation of coinciding autophagy/lysosomal phenotypes. Moreover, cell density-dependent changes in p62, Na^+^K^+^-ATPase and pS6 were detected in post-mitotic iPSC-derived neurons (Fig 4E and 4F and S8 Fig), which further supported the conclusion that cell cycle fluctuations are dispensable for triggering cellular adaptation to confluency.

### Allometric scaling of the plasma membrane and the nuclei is reflected in the Western blot analysis

**Fig 5:**
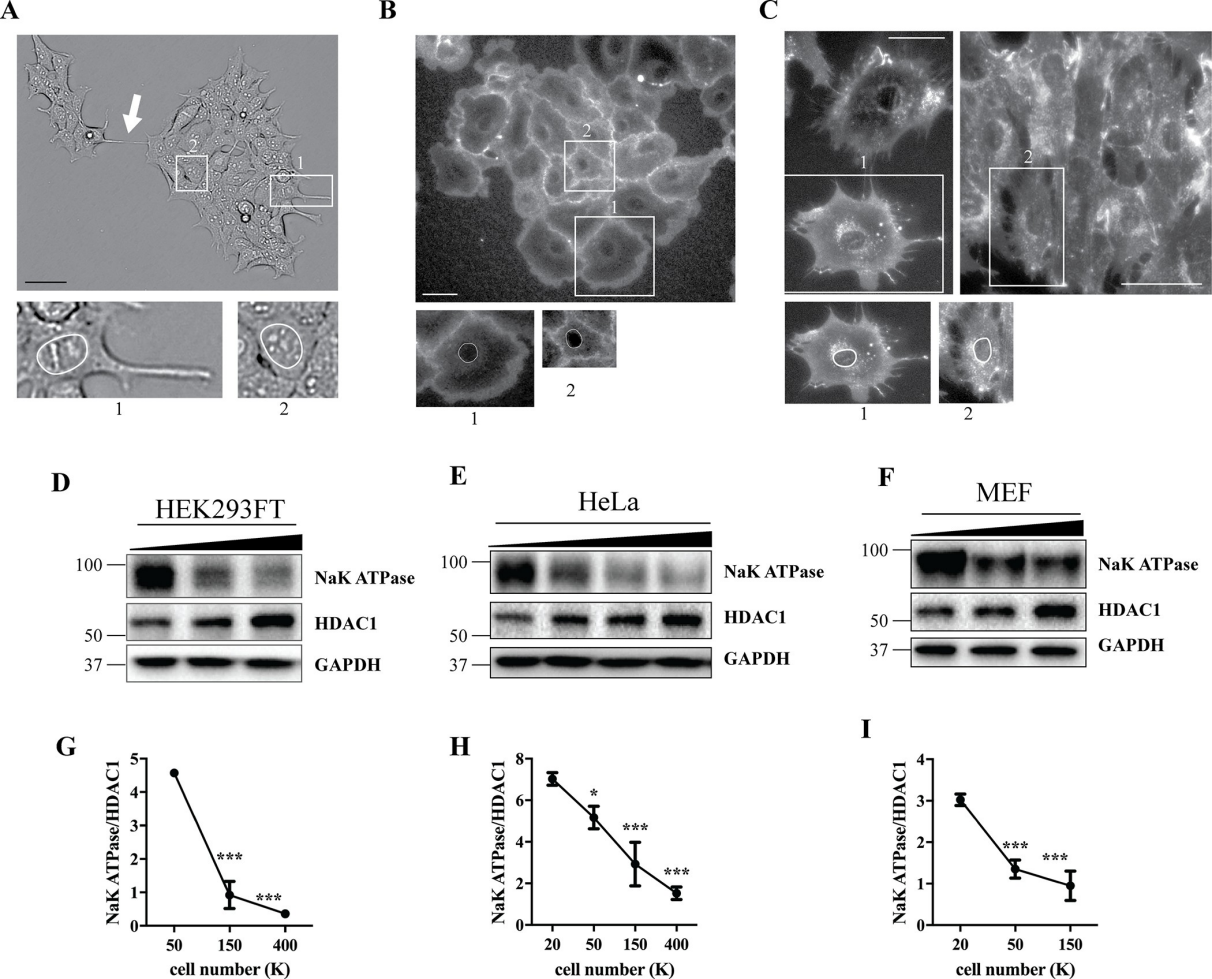
Allometric scaling of the plasma membrane and the nuclei is reflected in the Western blot analysis. 100K HEK 293FT (A), 50K HeLa (B) or 10K (C, left) and 200K (C, right) MEF cells were plated in 6 well plates, incubated for 2d, left unlabeled (A) or labeled with WGA (B,C) and imaged using bright field or fluorescent microscopy; 1, edge-cell (A,B) or cell in sparse conditions (C); 2, non-edge cell (A,B) or cell in crowded conditions (C); arrow in (A) points to a tunneling nanotube. Scale bar, 50 μm. (D-F) HEK 293FT, HeLa and MEF cells were plated at a range of densities. Cell lysates were analyzed by Western blotting using indicated antibodies. GAPDH was used as a loading control. (G-I) Western blot images were quantified and the Na^+^K^+^-ATPase to HDAC1 ratios plotted on the diagrams. N = 3; Line graph data are mean ± SD. *p<0.05, **p<0.01, ***p<0.001, relative to 50K (G), or 20K (H,I).

In addition to perturbations in signaling and metabolism and changes in corresponding protein markers, transitioning of the cells from sparse to crowded conditions has been associated with a reduction in cell size [5] (Fig 5A–5C), which entails allometric scaling of cellular compartments [25]. We then speculated that these morphometric changes might also be reflected on the biochemical level. To test this hypothesis, we first assessed the crowding behavior of HEK 293FT, HeLa and MEF cells. Interestingly, HEK 293FT cells were clustered in colonies where centrally positioned cells appeared small and round, whereas peripheral cells frequently extended long processes and connected with neighboring colonies via tunneling nanotubes (Fig 5A). These features in peripheral cells consumed substantial amounts of plasma membrane due to their high surface-to-volume ratio. Next, in WGA-labeled colonies of HeLa cells, surface of the peripheral cells was overall more spread out when compared to the cells confined within a colony (Fig 5B). Finally, MEF cells did not form colonies; in sparse conditions, they were mostly solitary, with spread out plasma membrane and multiple filopodia, while in confluent populations they became elongated, occupying smaller areas (Fig 5C). When the edge/sparse (1) and non-edge/crowded (2) cells were juxtaposed with magnification adjusted so that their nuclei were comparable in size, it was apparent that cell crowding led to a decrease in plasma membrane relative to the nuclei in all three cell lines (Fig 5A–5C). Western blot analysis was then used to quantify plasma membrane and nuclear markers Na^+^K^+^-ATPase and HDAC1, respectively, and this revealed a strong inverse correlation between the Na^+^K^+^-ATPase to HDAC1 ratio and cell density (Fig 5D–5I), in agreement with the observed morphometric changes. Similar trends were obtained for another plasma membrane resident–cadherin, and for a nuclear protein Lamin B1 (S9 Fig).

### Cell density affects experimental outcomes

**Fig 6:**
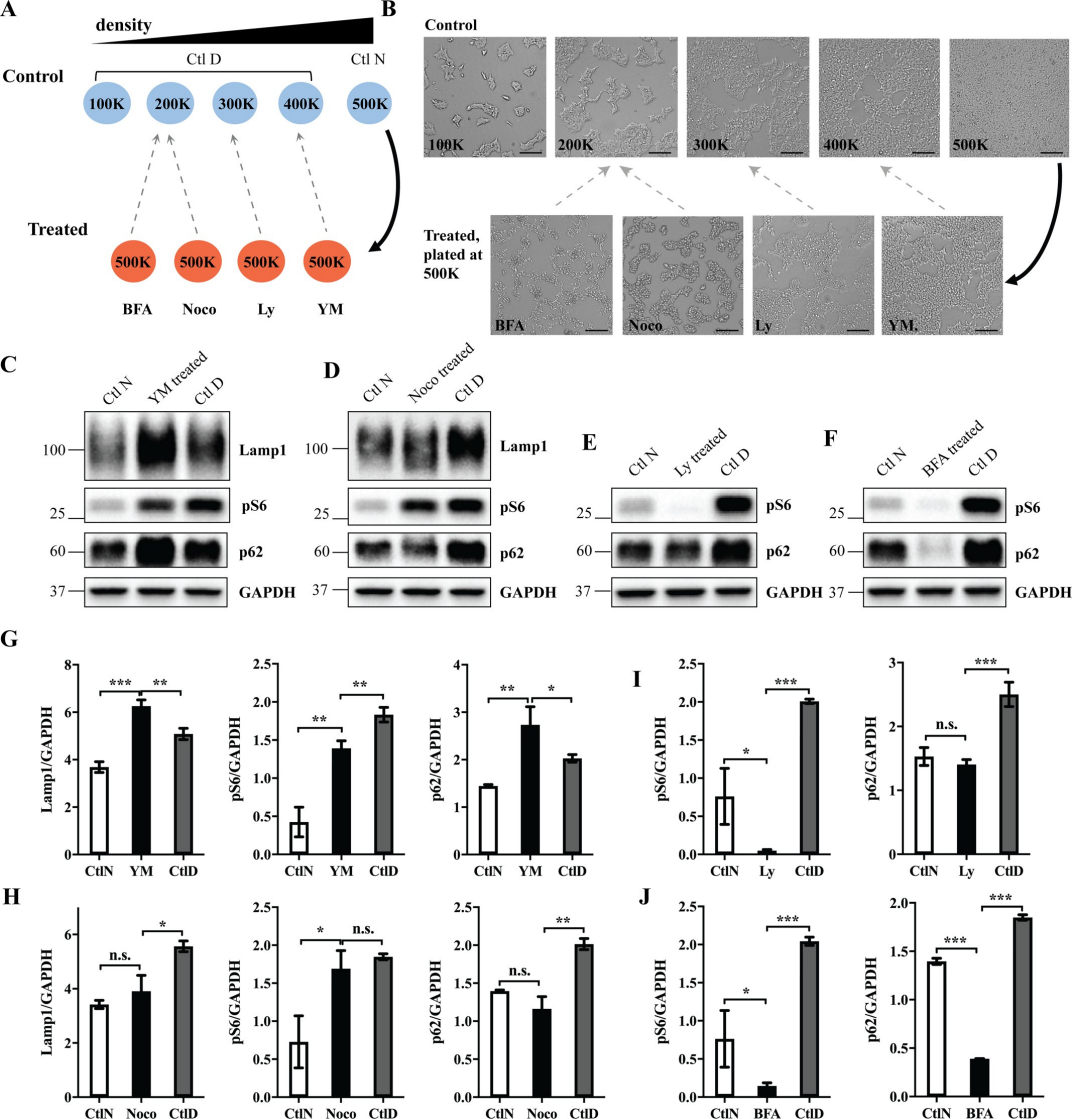
Cell density affects experimental outcomes. (A) HEK 293FT cells were plated in 6 well-plates according to the following scheme: 5 sets of control cells at a range of densities (100-500K cells per well) and 4 sets of cells at the highest density (500K cells per well) for treatment. All control cells were treated with the vehicle (control), and the remaining 4 sets were treated with 25 μM Ly294002 (Ly), 2.5 μg/μl Brefeldin A (BFA), 100 ng/ml nocodazole (noco) or 800 nM YM-201636 (YM), for the last 20h before lysis. Ctl N, control cells plated at the same number (500K) as the treated cells; Ctl D, control cells with matching cell density at the experimental endpoint. In classical experimental design, Ctl N is used as a control. (B) Cells were imaged before the lysis and matched with one of the controls based on cell density. Scale bar, 100 μm. (C-F) Cell lysates were analyzed by Western blotting using indicated antibodies. Ctl N and Ctl D were simultaneously compared with treated cells. GAPDH was used as a loading control. (G-J) Western blot images were quantified and the values normalized to GAPDH; Bar graph data are mean ± SD. *p<0.05, **p<0.01, ***p<0.001.

Together, our results show that cell density affects the expression levels of multiple proteins residing in different cellular compartments. The fact that agents used in experimental practice frequently alter confluency as a side effect led us to ask how this may affect experiments where treatments of interest lead to an undesired change in cell density relative to the control and the readout is a cell density-sensitive protein. To answer this question, five sets of control cells were plated at a range from 100K to 500K, and cells destined for treatment only at the highest number of 500K (Fig 6A). The latter were then treated under standard, previously established conditions [26–29] with four different drugs chosen based on their ability to affect cell density: brefeldin A, nocodazole, Ly294002 or YM-201636. As expected, all treatments eventually affected cell density. After overnight incubation, the cells were imaged and the treated cells were matched with one of the controls based on similarity in their densities at the end of the treatment (Fig 6B). In further analysis, treated cells were simultaneously compared with unmatched control that had identical starting cell number (Ctl N) and density-matched control (Ctl D) with similar endpoint density, as determined above (Fig 6C–6F). When cell density-responsive proteins pS6, p62 and Lamp1 were revealed by Western blot analysis, different results were recorded depending on the choice of control (Fig 6C–6J). Using the unmatched control (Ctl N) as opposed to a density-matched control (Ctl D) created (Fig 6H, center), erased (Fig 6I, right; 6H, left and right), inverted (Fig 6G, center), or affected significance (Fig 6G, left and right; 6I left and 6J left) of a difference in protein expression levels. The result was not affected significantly in one particular instance where p62 was visualized after treatment with Brefeldin A (Fig 6J right). This experiment demonstrates that cell density is a potent experimental variable, which may be a decisive parameter in experimental outcomes. Using density-matched controls may thus reduce the noise in data acquisition created by variations in cell density.

## Discussion

In this study, we show that markers of multiple cellular compartments adapt to variations in cell density. Autophagic/lysosomal proteins are regulated by mTOR and FAK signaling pathways that sense changes in nutrient availability and cell crowding in growing cell populations. The accompanying changes in the expression levels of plasma membrane and nuclear markers are consistent with allometric scaling of the respective compartments. We also demonstrate how selective adaptation of protein expression levels to cell density may confound experimental outcomes, and suggest a rational experimental design that minimizes cell density as a source of variability.

Previous research has shown that cell density plays a role in regulating biochemical and physiological properties of the cells, although the magnitude of density-dependent changes is debatable. Density-dependent alterations have been detected in the expression of over 2000 genes in human bone marrow stromal cells [30]. Nevertheless, global gene expression pattern was fairly stable in different states of confluence in oral carcinoma cell lines, despite the variability in the levels of individual genes [31]. In agreement with this study, we found no dramatic changes in the overall pattern of protein bands on polyacrylamide gels (Fig 1B; S1–S3 Figs) and in highly abundant proteins such as actin, calnexin and GAPDH (Fig 1D; S1–S3 Figs). However, at least a subset of autophagic, lysosomal, plasma membrane and nuclear markers, as well as components of the mTOR, FAK and Hippo signaling pathways displayed striking density-dependent variability in multiple cell types (Figs 1 and 3–5; S1–S3, S6 and S9 Figs). Interestingly, specific cellular functions are also highly sensitive to cell density. For instance, the rate of low-density lipoprotein metabolism in confluent tumor cells is ten times lower in comparison with preconfluent cells [32]. Moreover, the rate of clathrin-mediated endocytosis changes considerably in response to cell crowding [12]. It is likely that other functions are also affected, given that cell density controls signaling molecules with broad implications in cellular physiology, such as mTOR, FAK and Hippo [9, 10, 16]. More research is needed to further elucidate the purpose and mechanisms of the adaptive response to variation in cell density.

Our finding that autophagic and lysosomal markers respond to cell density is consistent with earlier studies showing density-dependent alterations in cellular proteolysis and lysosomal fractional volume [1, 3, 4]. In this study, we suggested a mechanism of this adaptation. Both media conditioning and cellular crowding controlled mTOR signaling pathway in growing cell populations (Fig 2C and 2D), in agreement with an earlier study [16]. This switch in mTOR signaling served as a trigger for accompanying perturbations of autophagic proteins (Fig 2A), as expected, since mTOR is the major regulator of autophagy [19]. The function of cell density-dependent changes in autophagy is not entirely clear. It is possible that an increase in autophagy in high-density conditions protects cells from dying upon overgrowth. It would thus be interesting to compare autophagic rates among cell lines which differ in their resistance to overgrowth. Further studies are needed to thoroughly investigate this issue.

Our study offers evidence that local crowding-dependent inactivation of FAK signaling pathway may be responsible for cell-to-cell variability in the lysosomal content in HEK 293FT cells (Fig 3). Similarly, altered FAK signaling has been linked to cell-to-cell variability in lipid composition [10]. Furthermore, we found that mTOR signaling is downregulated both upon chemical inhibition of FAK (Fig 3F and 3G) and in FAK-/- cells when compared to the wild-type cells (Fig 3H and 3I). This is in agreement with earlier reports demonstrating that FAK activates phosphoinositide 3-kinase [33] which, in turn, controls mTOR [34], as well as with studies documenting that mTOR is a downstream component in the FAK signaling pathway [35, 36]. Interestingly, the autophagic marker p62 was also downregulated upon FAK deletion or inactivation (Fig 3F–3I). Crowding-dependent alterations in FAK signaling may thus contribute to perturbations in autophagy through controlling mTOR signaling pathway, and perhaps through additional mechanisms. For instance, FAK has been recently implicated in autophagy since it can directly phosphorylate an autophagic protein beclin-1 [37], and in Src-selective autophagy through regulation of p70S6K [38]. Conversely, it is possible that mTOR-dependent alterations in autophagy lead to changes in focal adhesions, as suggested by Macleod and collaborators [39], which could create a feedback loop. Our experimental system using a five-point gradient of densities, as opposed to a simple low-to-high comparison, may provide insight into the order of events and coordination of different pathways in density-dependent signaling cascade. For instance, in HEK 293FT cells, FAK was inactivated at lower densities (Fig 3D), whereas mTOR inactivation occurred at higher densities (Fig 1D; S1–S3 Figs), suggesting that if the two proteins act in the same signaling cascade, FAK is likely to act upstream of mTOR. Hippo signaling pathway which controls contact inhibition of proliferation is yet another regulatory mechanism, which might contribute to cell density-associated phenotypes [9] (Fig 4A and 4B and S6 Fig). However, since some proteins responded to cell density in non-dividing neurons (Fig 4E and 4F), as well as in cells with stable cell cycle across all densities, such as HEK 293FT (Figs 1D, 4C and 4D), we concluded that fluctuations in proliferation rate were not a decisive factor in controlling the cellular response to population density in our experimental system. The fact that Hippo pathway was changed in all cell types with or without accompanying changes in the cell cycle suggest that other factors may contribute to cell cycle regulation under different cell density conditions in a cell type-dependent manner. One possibility is that the analyzed cell lines differ in mitochondrial energetics and the levels of mitochondrial protein Drp1 which couples cell density with cell cycle dynamics, as previously suggested. [40].

Cell-to-cell variation in lysosomal mass and inverse correlation between cell density and the levels of Lamp1 were detected only in one studied cell line and are thus unlikely to play a widespread role in adaptation to cell density. Nevertheless, this finding led us to an unexpected discovery that FAK regulates lysosomal proteins Lamp1 and cathepsin D, although the exact mechanism remains unclear. mTOR is unlikely to be involved in the lysosomal regulation as a part of the FAK signaling cascade, since its inactivation is expected to increase the levels of Lamp1 through TFEB-controlled transcription of lysosomal genes [24]. However, an interesting possibility arises from several recent publications. First, heterogeneity within the late endosomal/lysosomal population has been described with respect to the pH and molecular composition of individual compartments. [41]. Other studies have suggested that late endosomes/lysosomes might have alternative, non-degradative roles in integrin recycling, maintenance of focal adhesions, cell migration or signaling [42–44]. It is tempting to speculate that a subpopulation of lysosomes at the edge of HEK 293FT colonies plays a role in recycling and cell migration and is regulated by focal adhesions and FAK, as opposed to mTOR-mediated regulation of degradative lysosomes.

In this study, we identified cellular crowding as an important factor regulating autophagy. Moreover, uneven crowding and population context determined the lysosomal content of individual cells in HEK 293FT cell line (Fig 3A) and these local changes in Lamp1 abundance were quantifiable in Western blot analysis of the total cell population (Fig 3B and 3C). On the other hand, crowding-dependent morphological alterations in cell size and shape corresponded to biochemically detectable changes in at least some plasma membrane and nuclear markers, such as Na^+^K^+^-ATPase, cadherin, HDAC1 and Lamin B1 (Fig 5 and S9 Fig). This is important since studying allometric scaling of cellular compartments is typically restricted to morphometric disciplines and largely ignored in biochemical approaches. Our data emphasize that population context-dependent cell-to-cell variability and morphometric changes may be reflected in biochemical analysis. This might be of relevance for studying plasma membrane proteins such as ion channels and receptors, as well as transcription factors that reside in the nucleus. Other organelles’ markers might also respond to alterations in cell size and shape. Cytoskeletal elements are particularly attractive candidates due to increased demand for long-distance transport in larger cells. Since each cell line has its signature morphology, growing pattern and contact inhibition properties, it would be interesting to perform a comparative analysis of candidate proteins in different cell types.

The fact that cell confluency affects the expression levels of multiple proteins may have important implications for experimental method in cell biology and related disciplines. In particular, we demonstrated that a side effect of several drug treatments was a decrease in cell density (Fig 6B). If the experimental readout is one of the cell density-sensitive proteins, such as pS6, p62 and Lamp1, differences in confluency between control and treated cells might create noise, which in turn affects the accuracy of results (Fig 6C–6J). To improve accuracy in data acquisition, we proposed the use of density-matched controls as opposed to standard controls, which could minimize the impact of cell density as an undesired variable. The limitation of this approach is that it only considers cell density at the experimental endpoint and disregards the evolution of differential density during the experiment. Experimental procedures causing unintended changes in confluency extend beyond drug treatments. shRNA-mediated gene silencing or ectopic protein expression frequently affect the mitotic potential and/or survival of the cells. Genetically modified stable cell lines, different clones of the same line or primary cells extracted from multiple subjects/animals often proliferate at different rates. The concomitant alterations in protein expression may interfere not only with Western blot analysis, but also with other quantitative biochemical approaches.

Another corollary of the foregoing observations is that cell density-dependent phenotypes may interfere with data interpretation, since the detected alteration in confluence-sensitive proteins is typically interpreted as a direct consequence of given treatment, rather than as an unspecific effect secondary to alterations in cell density (S10 Fig). Interestingly, a recent survey reveals the prevailing scientists’ opinion that poor reproducibility in research has reached the crisis status [45, 46]. Recognizing that cell density figures in experimental outcomes may help reduce reproducibility issues that arise from overlooking cell density as a variable, as previously suggested [47]. Controlling, monitoring and reporting plated cell number and endpoint protein yields in scientific articles could thus reconcile at least some discrepancies in data obtained in different laboratories. However, even highly reproducible data derived from tissue culture models are often valued based on their translatability to *in vivo* models. It is unclear how cell density in monolayers relates to tissue organization *in vivo*, where cells reside in 3D space and engage in complex interactions with other cells and extracellular matrix. Controlling cell density *in vitro* may thus lead to an increase in accuracy and reproducibility, but still generate tissue culture-specific results.

In conclusion, our finding that cell confluency controls the expression of a subset of cellular proteins emphasizes that population context, in addition to cell-intrinsic factors, determines cellular physiology. Recognizing the importance of population factors can in turn advance scientific rigor in experimental practice.

## Materials and methods

### Cell culture, antibodies and reagents

HEK 293FT, HeLa, MEF, MEF FAK-/- and A431 cells were obtained from ATCC. All cell lines were maintained in DMEM supplemented with 10% FCS (Invitrogen). iPS cell line was generated from skin fibroblasts [48] and was previously characterized [49]. Differentiation of iPSCs into cortical glutamatergic neurons was performed using a previously described protocol [50]. Primary antibodies were purchased from the following manufacturers: Cell Signaling Technology [p-mTOR Ser2448 (5536S), mTOR (2983S), pS6 Ser235/236 (2211), S6 5G10 (2217), calnexin (2433), HDAC1 (2062S), p-cdc2 Y15 (4539T), pFAK Y397 (3283S), FAK (3285S), pan-cadherin (4068T), phospho-YAP Ser127 (4911S), YAP (14074T)], BD Transduction Laboratories^TM^ [p62 lck (610832)], Abcam [cathepsin D (ab75852)], Enzo Life Sciences [calnexin (SPA-865)]; Sigma Aldrich [p62 (P0067), actin AC-40 (A3853), MAP2 (M4403)], Millipore [GAPDH (MAB374)], DSHB [alpha1 Sodium Potassium ATPase a6f (AB_528092)], Santa Cruz [Lamp1 (sc-20011)], ProteinTech [Lamin B1 (12987-1-AP)], and BioLegend [βIII tubulin (801202)]. Reagents were obtained from Sigma-Aldrich [nocodazole (M1404), Y15/FAK inhibitor 14 (SML0837), Brefeldin A (B7651)], Cayman Chemical [Ly294002 (70920), YM-201636 (13576)], Thermo Fisher Scientific [SimplyBlue Safe Stain (LC6060); Wheat Germ Agglutinin (WGA) Alexa Fluor^TM^ 594 Conjugate (w11262)] and MedChem Express [Torin1 (HY-13003)].

### Cell plating, sample preparation, SDS-PAGE and Western blot analysis

Cell density gradients were designed for each cell line so that cell confluency for each point was visually comparable across all lines. For all experiments, cells were plated in tissue culture treated, uncoated 6-well plates and total volume of the media was adjusted to 2 ml per well. Incubation time was 47–49 h before termination of the experiments. Cells were washed three times in ice-cold PBS before lysis in RIPA buffer supplemented with protease and phosphatase inhibitor cocktail (Halt^TM^ Protease and Phosphatase Inhibitor Cocktail, PI78440, Thermo Fisher Scientific). Lysates were incubated for 50 min on ice with vortexing every 10 min, followed by centrifugation for 8 min on 10,000 x g. Protein concentration of the supernatants was determined using the bicinchoninic acid (BCA) assay (Sigma-Aldrich), and all lysates were adjusted to the same concentration. BCA assay was repeated, concentrations re-adjusted and 4 X Laemmli buffer supplemented with β-mercaptoethanol was added to 1 X final concentration. Equal protein loads were analyzed by SDS-PAGE and Western blotting. Proteins separated on polyacrylamide gels were revealed using SimplyBlue Safe Stain according to the manufacturer’s instruction. Immunolabeled proteins on membranes were visualized by chemiluminescence using the ChemiDoc System from Bio-Rad.

### Immunofluorescence, light and confocal microscopy

For immunofluorescence, cells were plated on gelatin-coated coverslips placed in 6-well plates. The immunofluorescence procedure was described previously [51]. The coverslips were mounted in DAPI Fluoromount-G from Southern Biotech. Images were obtained on a Leica DMI4000B inverted confocal microscope.

For bright field microscopy, cells were prepared as above and imaged live using Leica DMI3000B microscope. Where indicated, membranes were visualized using fluorescently labeled WGA, according to manufacturers’ instructions.

### Data analysis and statistics

Quantitative analysis of Western blots was performed using Fiji software. The data were processed in GraphPad Prism 7 software. Significance was determined using one-way analysis of variance/Dunnett’s Multiple Comparison Test and unpaired Student’s t test.

## Supporting information

S1 Fig Autophagic proteins, mTOR signaling and cathepsin D are sensitive to population density in A431 cells. (A) A431 cells plated at a range of densities were incubated for two days and imaged by light microscopy. 1, 30K; 2, 150K; 3, 400K; 4, 800K; 5, 1200K. Scale bar 100 μm. (B) Cells were lysed and equal amounts of proteins were separated by SDS PAGE, followed by visualization of the proteins by SimplyBlue; (C) pH of the media was determined before the cell lysis; (D) Cell lysates were analyzed by Western blotting using indicated antibodies; (E-G) Western blot images were quantified and the values normalized to GAPDH, unless indicated otherwise. N = 3; Line graph data are mean ± SD. *p<0.05, **p<0.01, ***p<0.001, relative to 1.
(TIF)

S2 Fig Autophagic proteins and mTOR signaling are sensitive to population density in HeLa cells. (A) HeLa cells plated at a range of densities were incubated for two days and imaged by light microscopy. 1, 20K; 2, 50K; 3, 150K; 4, 400K; 5, 800K. Scale bar 100 μm. (B) Cells were lysed and equal amounts of proteins were separated by SDS PAGE, followed by visualization of the proteins by SimplyBlue; (C) pH of the media was determined before the cell lysis; (D) Cell lysates were analyzed by Western blotting using indicated antibodies; (E-G) Western blot images were quantified and the values normalized to GAPDH, unless indicated otherwise. N = 3, except for p62, actin (N = 4) and GAPDH (N = 5); Line graph data are mean ± SD. *p<0.05, **p<0.01, ***p<0.001, relative to 1.
(TIF)

S3 Fig Markers of autophagy, mTOR signaling and cathepsin D are sensitive to cell confluence in MEF cells. (A) MEF cells plated at a range of densities were incubated for two days and imaged by light microscopy. 1, 20K; 2, 50K; 3, 150K; 4, 400K; 5, 800K. Scale bar 100 μm. (B) Cells were lysed and equal amounts of proteins were separated by SDS PAGE, followed by visualization of the proteins by SimplyBlue; (C) pH of the media was determined before the cell lysis; (D) Cell lysates were analyzed by Western blotting using indicated antibodies; (E-G) Western blot images were quantified and the values normalized to GAPDH, unless indicated otherwise. N = 3; Line graph data are mean ± SD. *p<0.05, **p<0.01, ***p<0.001, relative to 1.
(TIF)

S4 Fig Lamp1 within colonies of HEK 293FT cells is more abundant in edge-cells as compared to the non-edge cells. HEK 293FT cells were plated at 100K on coverslips placed in a 6 well plate, incubated for 2 days, fixed and stained against Lamp1; DAPI was used to visualize nuclei. Scale bar, 20 μm.
(TIF)

S5 Fig Lamp1 does not depend on population context in A431 cells. (A) A431 cells were plated at a range of densities and incubated for two days. Cell lysates were analyzed by Western blotting using indicated antibodies. GAPDH was used as a loading control. (B) Western blot images were quantified and the values normalized to GAPDH. Plated number of cells: 1, 30K; 2, 150K; 3, 400K; 4, 800K; 5, 1200K. Scale bar, 20 μm. (C) 100K A431 cells were plated on coverslips placed in a 6 well plate, incubated for 2 days, fixed and stained against Lamp1. DAPI was used to visualize nuclei. Scale bar, 20 μm.
(TIF)

S6 Fig Hippo signaling depends on cell density in A431, HeLa and MEF cells. (A, C, E) Cells were plated at a range of densities and incubated for two days. Cell lysates were analyzed by Western blotting using indicated antibodies. (B, D, F) Western blot images were quantified and the values normalized to total YAP. Plated number of cells: for A431 as in S1 Fig; for HeLa as in S2 Fig; for MEF as in S3 Fig. Line graph data are mean ± SD. *p<0.05, **p<0.01, ***p<0.001, relative to point 1.
(TIF)

S7 Fig Cell cycle dynamics changes with population density in MEF and HeLa cells. MEF (A) and HeLa (C) cells were plated at a range of densities, incubated for 2 days, lysed and analyzed by Western blotting using indicated antibodies. GAPDH was used as a loading control. Plated cell number: 1, 20K; 2, 50K; 3, 150K; 4, 400K; 5, 800K. (B,D) Western blot images were quantified and the values normalized to GAPDH. N = 3; Line graph data are mean ± SD. *p<0.05, **p<0.01, ***p<0.001, relative to 1.
(TIF)

S8 Fig Quality of cortical motor neurons. Neuronal cultures were imaged by light microscopy after transduction by EGFP lentivirus (A, B) and after immunofluorescence using MAP2 antibody (C).
(TIF)

S9 Fig Allometric scaling of the plasma membrane and the nuclei is reflected in the Western blot analysis of cadherin and Lamin B1. (A, C) HeLa and MEF cells were plated at a range of densities and incubated for two days. Cell lysates were analyzed by Western blotting using indicated antibodies. GAPDH was used as a loading control. (B, D) Western blot images were quantified and the values normalized to GAPDH. Plated number of cells: for HeLa as in panel A1 in S2 Fig; for MEF as in panel A1 in S3 Fig. Line graph data are mean ± SD. *p<0.05, **p<0.01, ***p<0.001, relative to point 1.
(TIF)

S10 Fig Errors in data interpretation due to unintended changes in cell density. Drug treated or genetically modified cells, stably transformed cell lines or different clones of the same cell line often differ in proliferation rate or viability when compared to corresponding controls, which ultimately results in different cell densities (1.). This is followed by cell density-dependent variation in a subset of proteins/cellular functions (2.). If the cell density alterations are not considered, the detected system variation may be incorrectly interpreted as a direct effect of given intervention (bold arrow).
(TIF)
