# Supplementary figures and images for: Fluctuations in cell density alter protein markers of multiple cellular compartments, confounding experimental outcomes
Source: PLoS One. 2019 Feb 4;14(2):e0211727. doi: 10.1371/journal.pone.0211727 (PMC6361456; doi:10.1371/journal.pone.0211727)

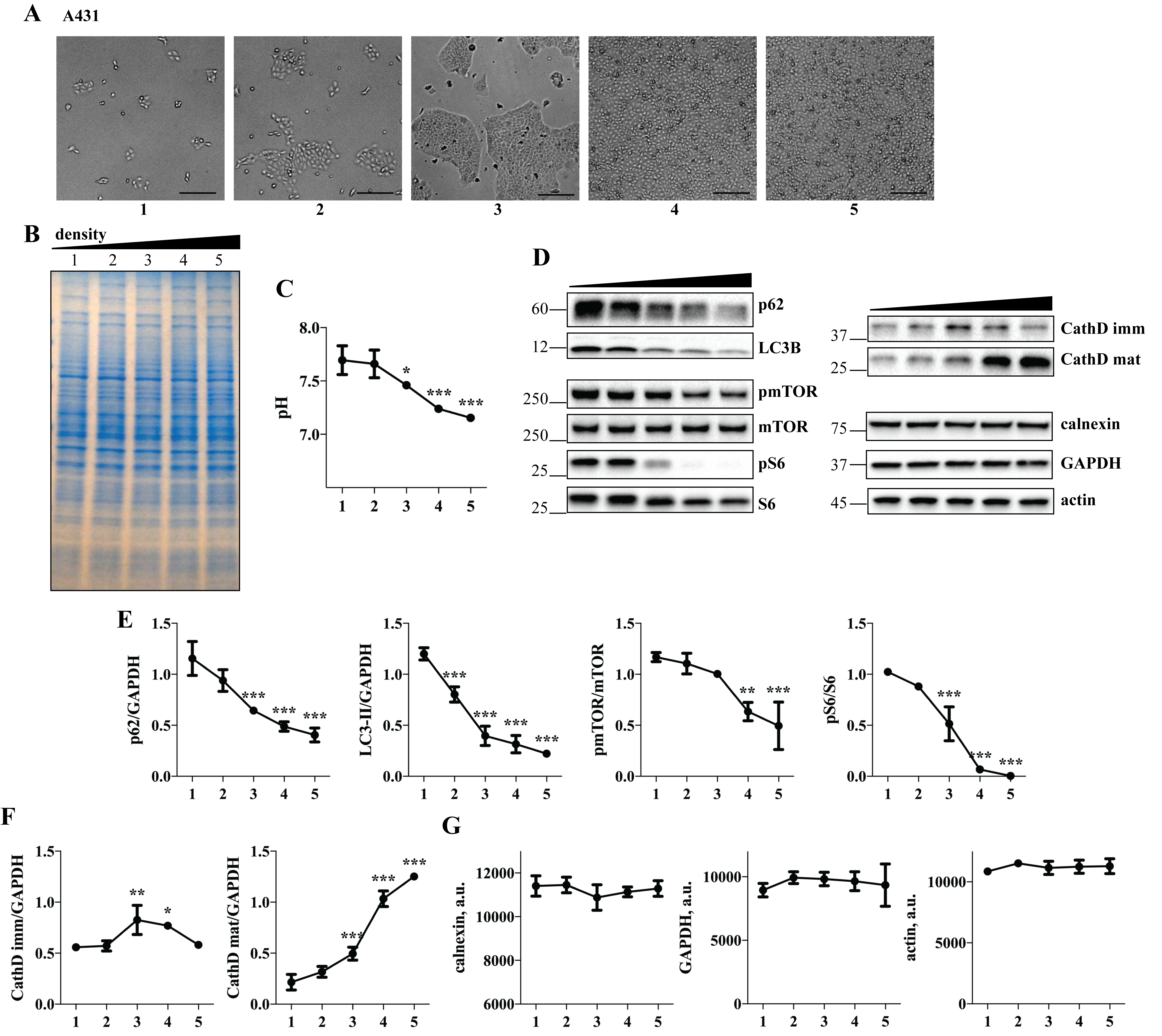

Supplement: S1 Fig — (A) A431 cells plated at a range of densities were incubated for two days and imaged by light microscopy. 1, 30K; 2, 150K; 3, 400K; 4, 800K; 5, 1200K. Scale bar 100 μm. (B) Cells were lysed and equal amounts of proteins were separated by SDS PAGE, followed by visualization of the proteins by SimplyBlue; (C) pH of the media was determined before the cell lysis; (D) Cell lysates were analyzed by Western blotting using indicated antibodies; (E-G) Western blot images were quantified and the values normalized to GAPDH, unless indicated otherwise. N = 3; Line graph data are mean ± SD. *p<0.05, **p<0.01, ***p<0.001, relative to 1. (TIF) [file pone.0211727.s001.tif]

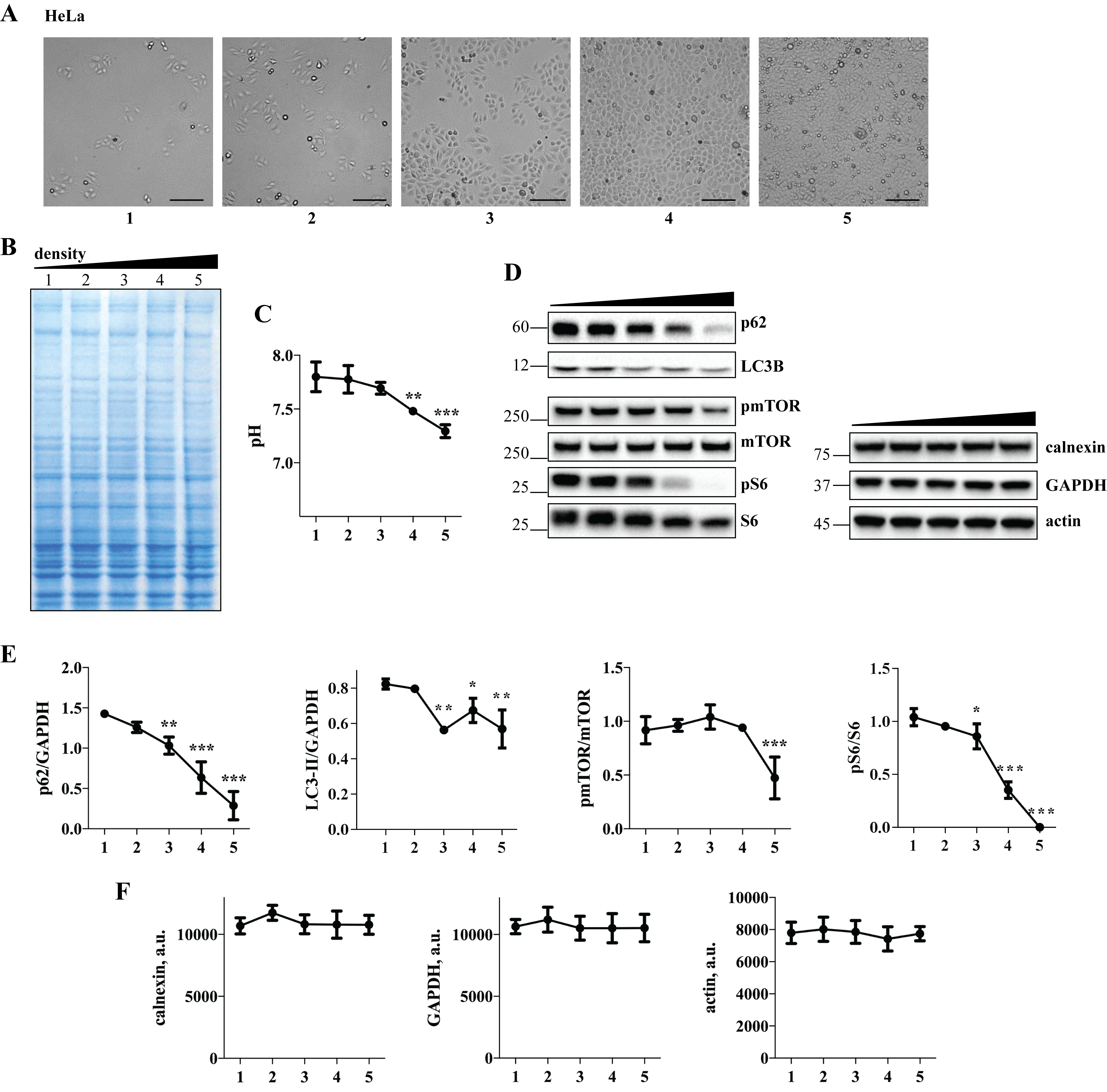

Supplement: S2 Fig — (A) HeLa cells plated at a range of densities were incubated for two days and imaged by light microscopy. 1, 20K; 2, 50K; 3, 150K; 4, 400K; 5, 800K. Scale bar 100 μm. (B) Cells were lysed and equal amounts of proteins were separated by SDS PAGE, followed by visualization of the proteins by SimplyBlue; (C) pH of the media was determined before the cell lysis; (D) Cell lysates were analyzed by Western blotting using indicated antibodies; (E-G) Western blot images were quantified and the values normalized to GAPDH, unless indicated otherwise. N = 3, except for p62, actin (N = 4) and GAPDH (N = 5); Line graph data are mean ± SD. *p<0.05, **p<0.01, ***p<0.001, relative to 1. (TIF) [file pone.0211727.s002.tif]

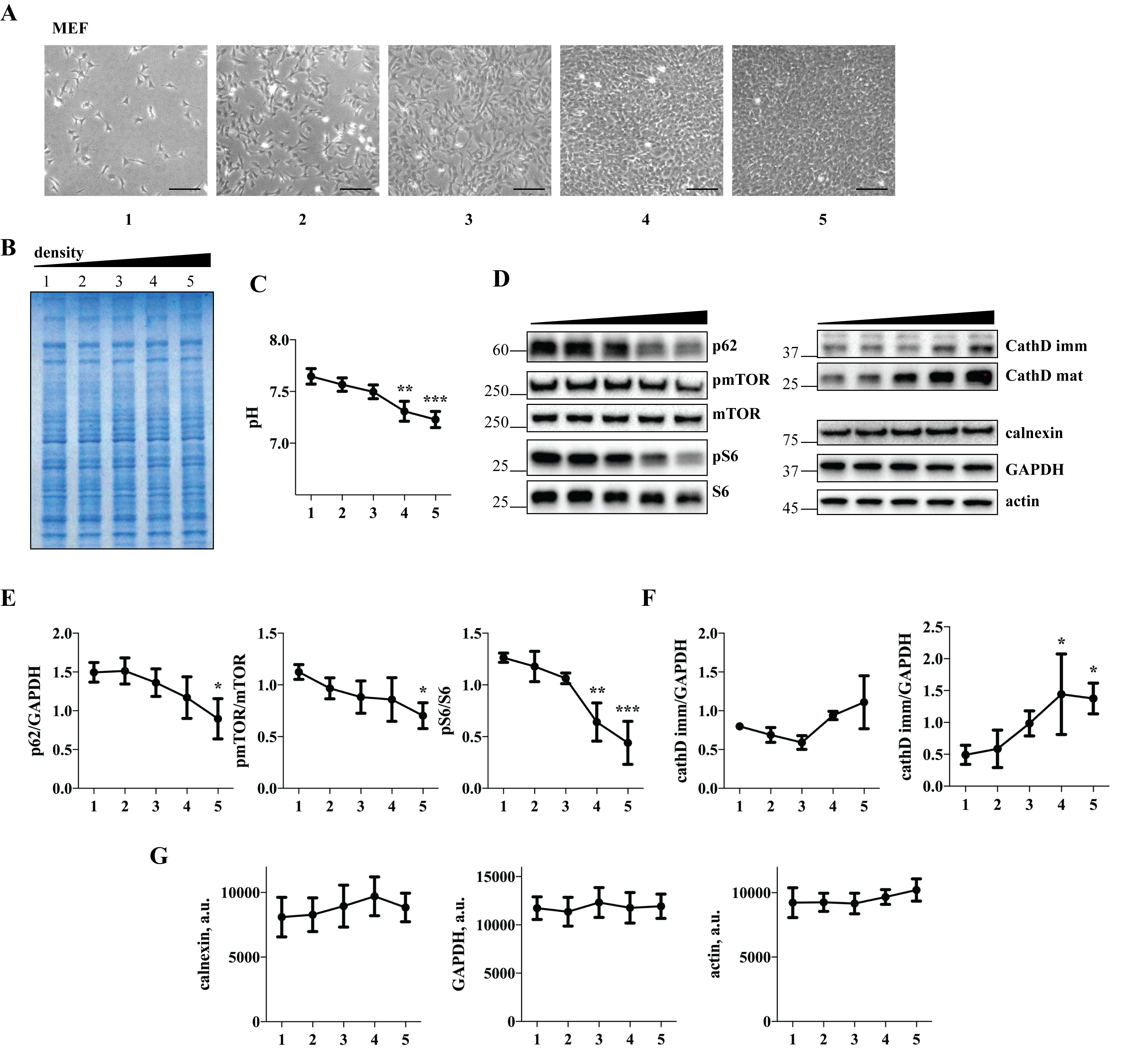

Supplement: S3 Fig — (A) MEF cells plated at a range of densities were incubated for two days and imaged by light microscopy. 1, 20K; 2, 50K; 3, 150K; 4, 400K; 5, 800K. Scale bar 100 μm. (B) Cells were lysed and equal amounts of proteins were separated by SDS PAGE, followed by visualization of the proteins by SimplyBlue; (C) pH of the media was determined before the cell lysis; (D) Cell lysates were analyzed by Western blotting using indicated antibodies; (E-G) Western blot images were quantified and the values normalized to GAPDH, unless indicated otherwise. N = 3; Line graph data are mean ± SD. *p<0.05, **p<0.01, ***p<0.001, relative to 1. (TIF) [file pone.0211727.s003.tif]

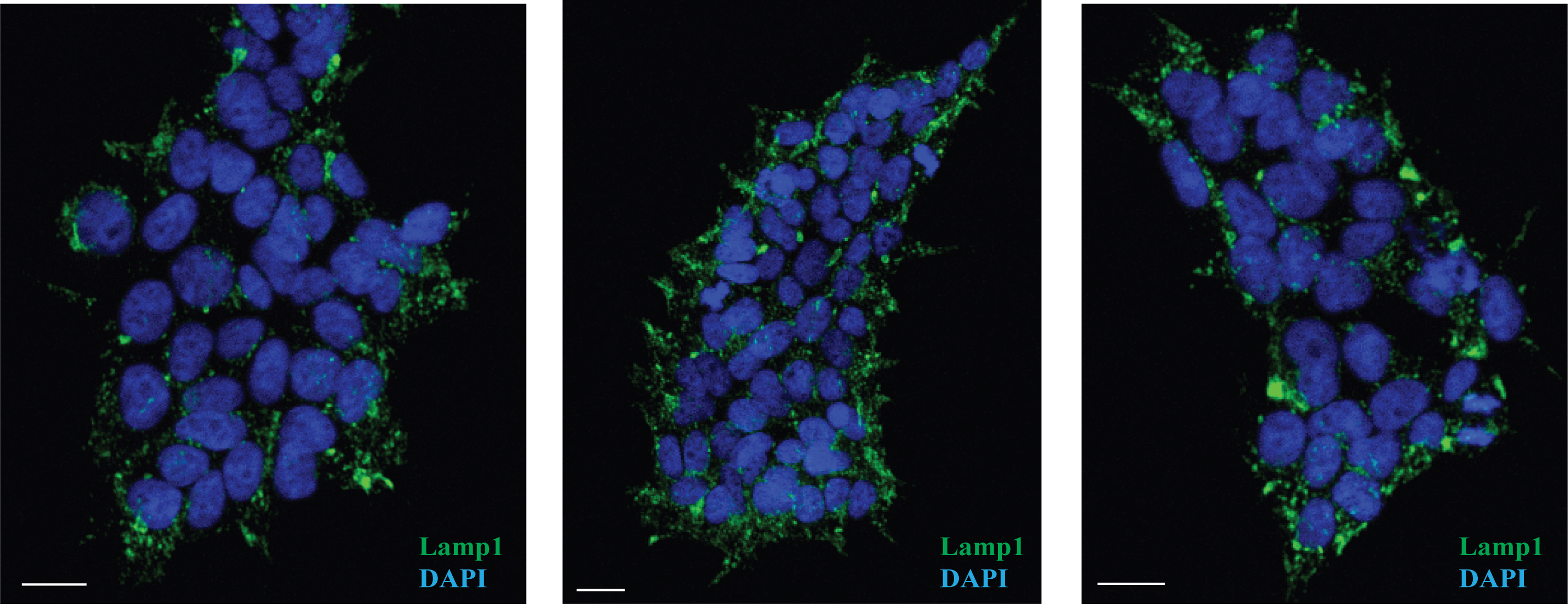

Supplement: S4 Fig — HEK 293FT cells were plated at 100K on coverslips placed in a 6 well plate, incubated for 2 days, fixed and stained against Lamp1; DAPI was used to visualize nuclei. Scale bar, 20 μm. (TIF) [file pone.0211727.s004.tif]

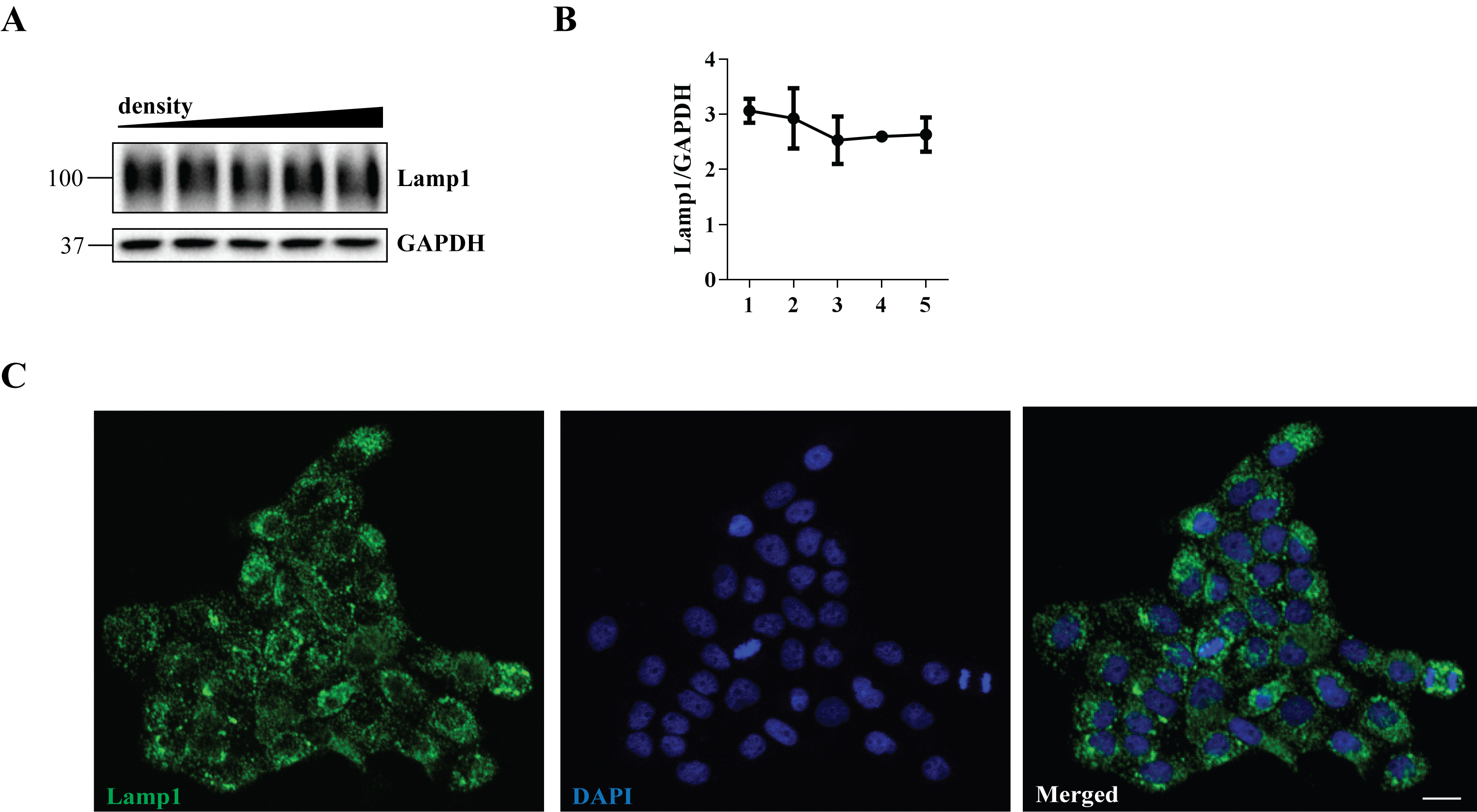

Supplement: S5 Fig — (A) A431 cells were plated at a range of densities and incubated for two days. Cell lysates were analyzed by Western blotting using indicated antibodies. GAPDH was used as a loading control. (B) Western blot images were quantified and the values normalized to GAPDH. Plated number of cells: 1, 30K; 2, 150K; 3, 400K; 4, 800K; 5, 1200K. Scale bar, 20 μm. (C) 100K A431 cells were plated on coverslips placed in a 6 well plate, incubated for 2 days, fixed and stained against Lamp1. DAPI was used to visualize nuclei. Scale bar, 20 μm. (TIF) [file pone.0211727.s005.tif]

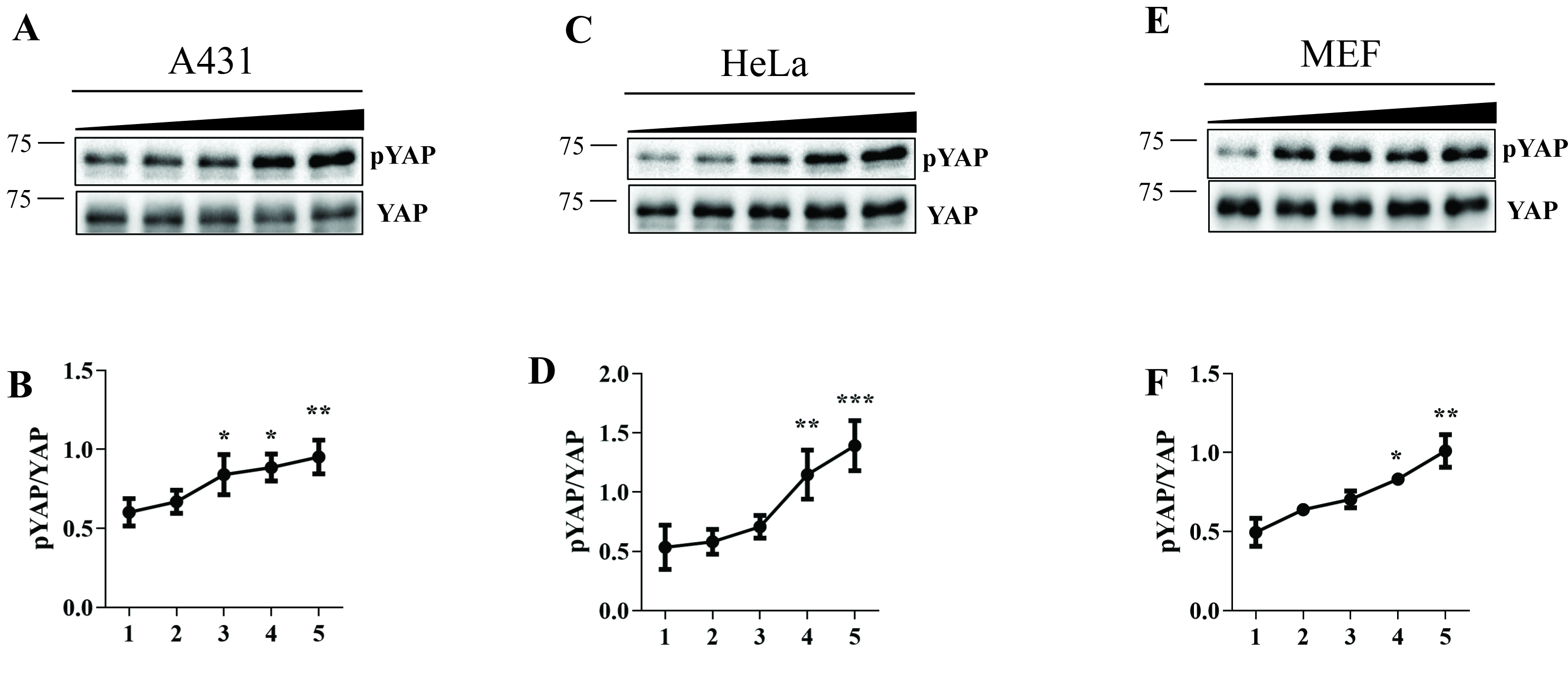

Supplement: S6 Fig — (A, C, E) Cells were plated at a range of densities and incubated for two days. Cell lysates were analyzed by Western blotting using indicated antibodies. (B, D, F) Western blot images were quantified and the values normalized to total YAP. Plated number of cells: for A431 as in S1 Fig; for HeLa as in S2 Fig; for MEF as in S3 Fig. Line graph data are mean ± SD. *p<0.05, **p<0.01, ***p<0.001, relative to point 1. (TIF) [file pone.0211727.s006.tif]

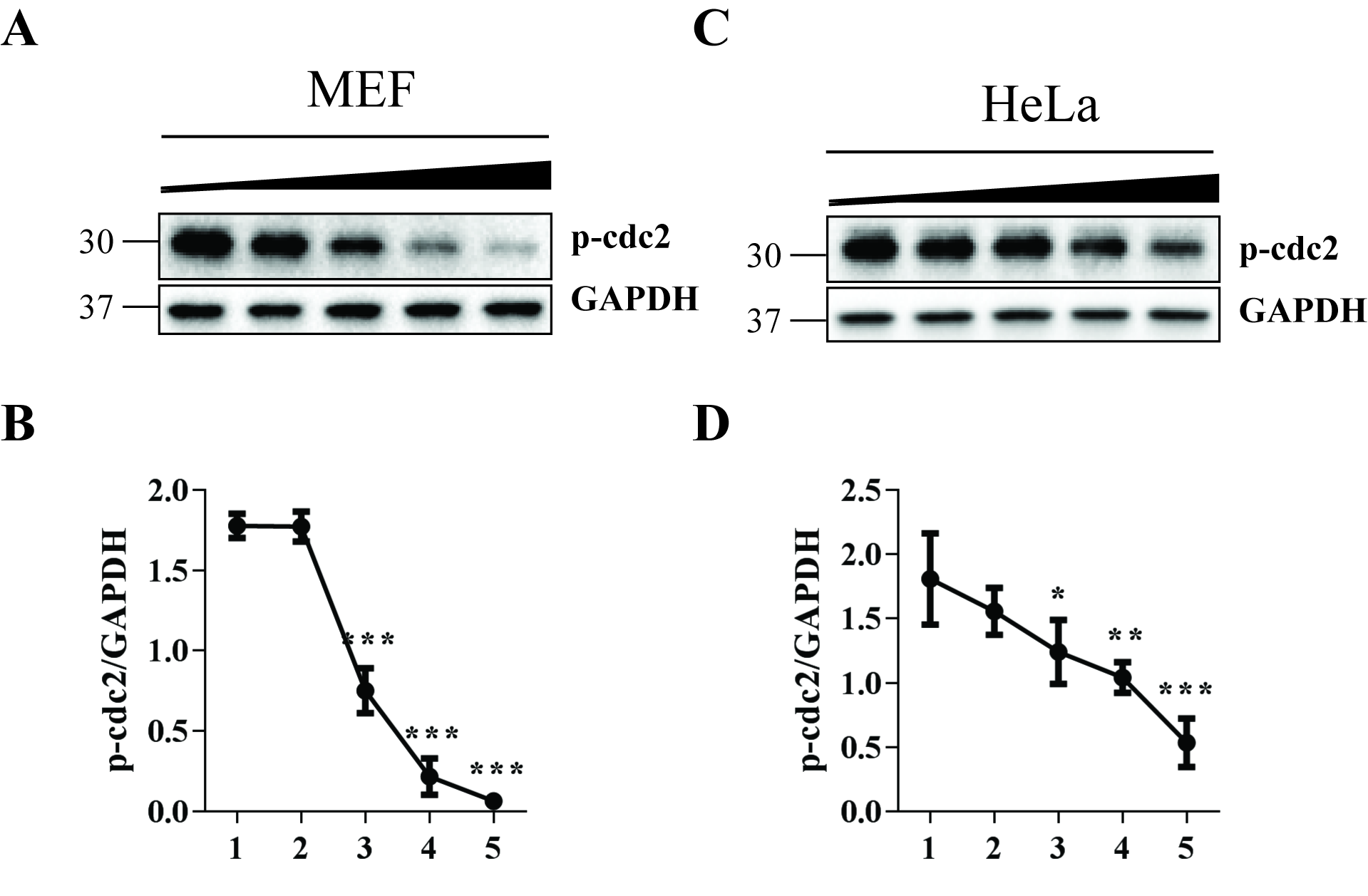

Supplement: S7 Fig — MEF (A) and HeLa (C) cells were plated at a range of densities, incubated for 2 days, lysed and analyzed by Western blotting using indicated antibodies. GAPDH was used as a loading control. Plated cell number: 1, 20K; 2, 50K; 3, 150K; 4, 400K; 5, 800K. (B,D) Western blot images were quantified and the values normalized to GAPDH. N = 3; Line graph data are mean ± SD. *p<0.05, **p<0.01, ***p<0.001, relative to 1. (TIF) [file pone.0211727.s007.tif]

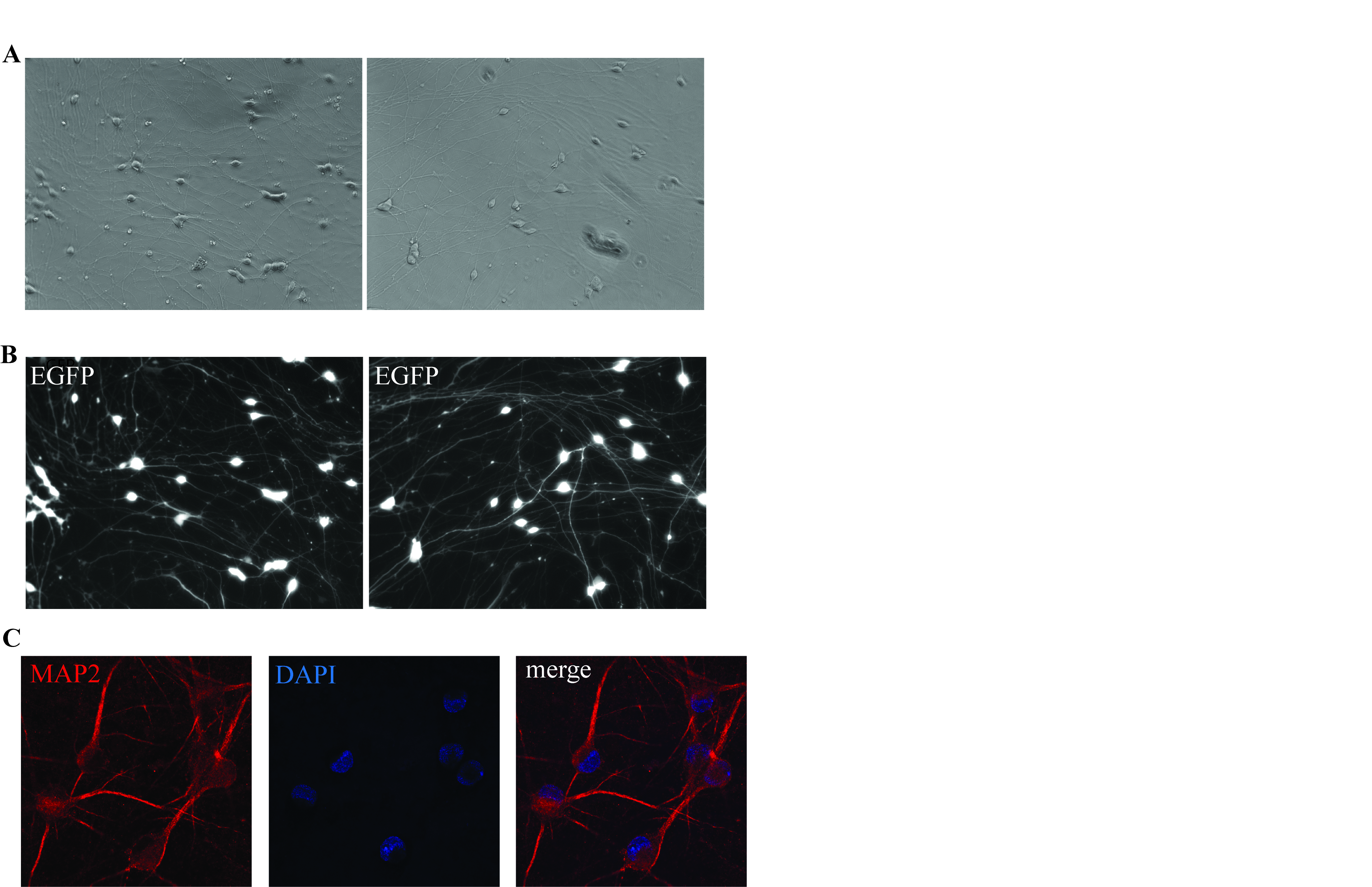

Supplement: S8 Fig — Neuronal cultures were imaged by light microscopy after transduction by EGFP lentivirus (A, B) and after immunofluorescence using MAP2 antibody (C). (TIF) [file pone.0211727.s008.tif]

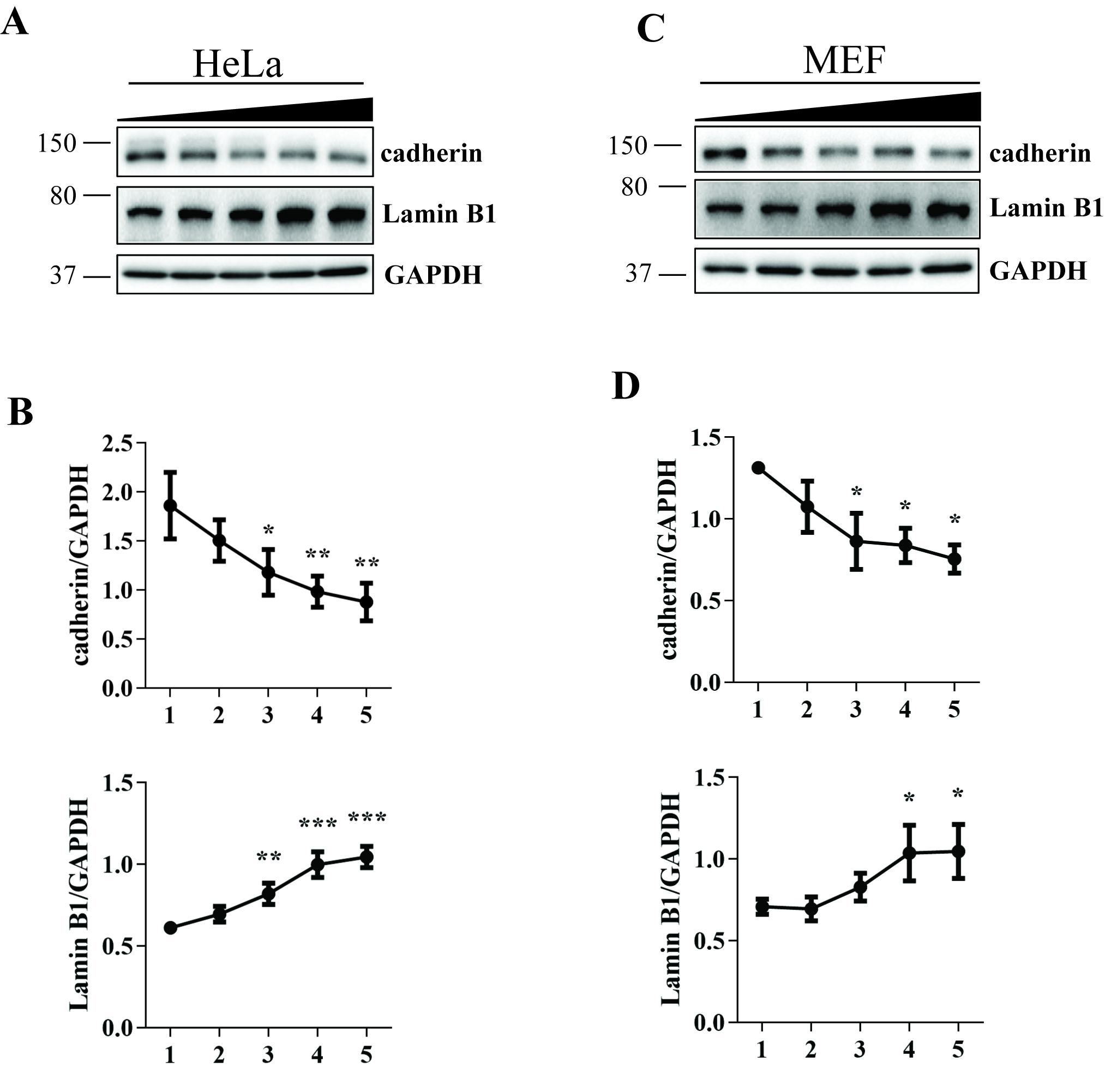

Supplement: S9 Fig — (A, C) HeLa and MEF cells were plated at a range of densities and incubated for two days. Cell lysates were analyzed by Western blotting using indicated antibodies. GAPDH was used as a loading control. (B, D) Western blot images were quantified and the values normalized to GAPDH. Plated number of cells: for HeLa as in panel A1 in S2 Fig; for MEF as in panel A1 in S3 Fig. Line graph data are mean ± SD. *p<0.05, **p<0.01, ***p<0.001, relative to point 1. (TIF) [file pone.0211727.s009.tif]

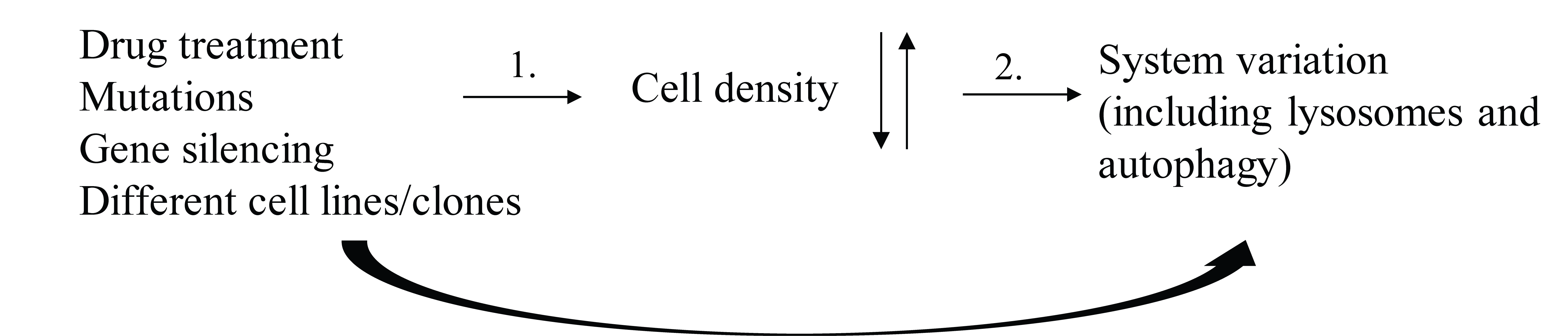

Supplement: S10 Fig — Drug treated or genetically modified cells, stably transformed cell lines or different clones of the same cell line often differ in proliferation rate or viability when compared to corresponding controls, which ultimately results in different cell densities (1.). This is followed by cell density-dependent variation in a subset of proteins/cellular functions (2.). If the cell density alterations are not considered, the detected system variation may be incorrectly interpreted as a direct effect of given intervention (bold arrow). (TIF) [file pone.0211727.s010.tif]
